# Induced Aberrant Organisms with Novel Ability to Protect Intestinal Integrity from Inflammation in an Animal Model

**DOI:** 10.3390/nu9080864

**Published:** 2017-08-11

**Authors:** Helieh S. Oz

**Affiliations:** Department of Physiology, Internal Medicine, College of Medicine, University of Kentucky Medical Center, Lexington, KY 40536-0298, USA; hoz2@email.uky.edu

**Keywords:** infection, gut inflammation, immunosuppression, poultry, coccidiosis, aberrant organisms

## Abstract

Robust and balanced gut microbiota are required to support health and growth. Overgrowth of gut microbial or pathogens can change ecosystem balance, and compromise gut integrity to initiate gastrointestinal (GI) complications. There is no safe and effective modality against coccidiosis. Antibiotic additives routinely fed to food animals to protect against infection, are entered into the food chain, contaminate food products and pass to the consumers. Hypothesis: induced aberrant organisms possess distinct ultrastructure and are tolerated by immunodeficient-animals yet are non-pathogenic, but immunogenic in various strains of chicks to act as a preventive (vaccine) and eliminating the needs for antibiotic additives. **Methods:** cyclophosphamide-immunodeficient and immune-intact-chicks were inoculated with induced aberrant or normal *Coccidal*-organisms. Immune-intact-chicks were immunized with escalating-doses of organisms. **Results:** Aberrant organisms showed distinct ultrastructure with 8-free-sporozoites which lacked sporocysts walls and veils. Immunodeficient-chicks inoculated with normal-organisms developed severe GI complications but tolerated aberrant-organisms (*p* < 0.001) while they had no detectable antibodies. Naïve-animals challenged with a pathogenic-dose showed GI complications, bloody diarrhea, severe lesions and weight loss. Immune-intact-animals immunized with aberrant forms were protected against high dose normal-pathogenic-challenge infection and gained more weight compared to those immunized with normal-organisms (*p* < 0.05). **Conclusions:** Aberrant organisms possess a distinct ultrastructure and are tolerated in immunodeficient-chicks, yet provide novel immune-protection against pathogenic challenges including diarrhea, malnutrition and weight loss in immune-intact-animals to warrant further investigations toward vaccine production.

## 1. Introduction

Robust and balanced gut microbiota are required to support health and growth. Application of certain medications and chemicals including antibiotics and immunmodulators can alter this delicate balance in digestive tracts and promote the state of disease [1]. Antibiotics are designed and required modality to abolish infectious pathogenic elements and to cure patients. Yet, antibiotics underlay a dual conundrum, as they can destroy the normal gut microbiome and promote growth of pathogenic organisms to cause dysbiosis. Further, altered permeability damages the gut mucosa and prone individuals to diarrhea and gastroenteritis.

Cyclophoshamide (CY) is an alkylating agent and a potent immunosuppressant compound used in humans against autoimmune diseases and cancer chemotherapy. CY affects both T and B cells; however, B cells have a slower rate of recovery [2,3]. CY is metabolized in hepatic cells to yield active substances and to exert cytotoxic effects on certain immune cells [4]. The cytotoxic effect is due to drug cross-link DNA, which can immediately destroy affected cells, render them susceptible to cell death during mitosis, or permit normal cellular activities if DNA repair occur. Indeed, CY-chemotherapy disrupts the gut epithelial barrier, and causes the gut to bypass certain bacteria. Bacteria gather in lymphoid tissue just outside the gut and spur generation of T helper 1 and T helper 17 cells that migrate to the tumor vicinity and destroy tumor cells [5]. In birds, immunoglobulin synthesis and antibody production depend upon the integrity of bursa of fabricius lymphoid organ which is located on the dorsal side of rectal “cloaca”. CY-treatment on the first three days after birth is reported to severely depress antibody production as animals fail to produce antibodies against antigenic challenges up to 7–11 weeks, similar to combined bursectomized and in ova X-irradiation [6]. In addition, CY causes significant decreases in lymphoid population in thymus [2]. The splenic structure and preferral lymphoid organs lost germinal and plasma cells. The variation in the action and the outcome reported in different studies are due to the doses, the length and the time for initiation of CY-administration. 

Coccidia are highly host specific transmissible Apicomplexan organisms which mainly attack gut mucosa and compromise the immune system to trigger gastrointestinal inflammation, infectious diarrhea, loss of function and morbidity in humans and animals. In contrast, *Toxoplasma,* another member of the family Apicomplexan, is a ubiquitous organism which infects every organ and cell in animals and humans to cause toxoplasmosis [7]. Coccidiosis is one of the most important communicable pathogenic diseases in the food animals industry. Additionally, *Coccidia* predispose infected animals to other pathogens like *Clostridia* and more severe necrotic enteritis [8]. There is no safe and effective therapeutic modality or vaccine to protect against the infection [9]. 

Antibiotic additives are routinely fed to poultry and livestock as a common practice to protect against the infection and weight loss. These additives contaminate egg, meat, bone and milk products which are transferred into the food chain and consumed with predicted complications. For instance, quinolones are commonly prescribed in patients while used in diets for the poultry industry to increase weight gain and growth [10,11]. Quinolone residues are detected in 50% of eggs at higher concentrations above the limits for edible tissues established by the regulatory agencies, including United States Department of Agriculture [11]. Animal products contaminated with antibiotic residues create a great concern about possible side effects in consumers such as allergies [12] and potential for antibiotic-resistant microbials. 

Coccidiosis causes great economic loss and morbidity by reduction in food intake, weight gain and egg production, and also affects the value of meat quality by decreasing feed conversion, maldigestion and malabsorption to lead in mortality [3]. The annual cost of coccidiosis in poultry production has been estimated at $800 million in the USA [13,14], chiefly for anticoccidial drugs which are commonly used to control the infection and to improve weight gain. The constant addition of medications in food animal diets has been a profitable and effective tool against the disease outbreak, but there are drawbacks including development of drug resistance and potential health side effects in consumers. The live vaccine, Coccivac, is a mixture of seven species of poultry *Coccidia* which has been utilized for over five decades in the USA [15]. The animals recover the infection from vaccines and develop immunity which lasts days to weeks. The disadvantages of this vaccine include: poor feed conversion to weight gain; several weeks are required to develop a solid immunity; possibility of spreading infection; difficulties in administering the vaccine and managing the animals. Other possible vaccines in experimental stages are attenuated strains including serially transferred *Eimeria tenella (E. tenella)* into chorio-allantoic chick embryos [16]. 

Ever since its discovery (Fantham and Porter 1900), Eimeria [17] have been described as organisms (oocysts) with four sporocysts, each containing two sporozoites. By utilizing purification procedures, aberrant forms of different Eimerias were induced which matured to contain 8-free-sporozoites with no protective sporocyst walls, as were confirmed by light microscopy [18]. The aberrant organisms proved to be less pathogenic than the normal form in inbred Leghorn-chicks, but similarly immunogenic. The hypothesis of this investigation was: induced aberrant organisms possess a distinct ultrastructure and are tolerated by immunodeficient-animals, yet are non-pathogenic but immunogenic in various strains of chicks to act as preventive (vaccine) and eliminating the need for antibiotic additives. This investigation reports the ultrastructural formation of these novel organisms and further compares their pathogenecity to normal forms in immunodeficient and susceptible animals. In addition, immunogenicity of these aberrant forms is examined and compared to normal organisms utilizing two diverse inbred chicks, Rhode Island Red and New Hampshire strains.

## 2. Materials and Methods

### 2.1. Ethical Guidelines for the Use of Animals 

This investigation was conducted according to the guidelines of the National Institute of Health and the International and the American Pain Associations. All animal procedures were approved by the University of Kentucky Institutional Animal Care and Use Committee (IACUC) and Institutional Biosafety Committee.

### 2.2. Animals

One-day-old (neonate) specific pathogen-free inbred Rhode Island Red and inbred New Hampshire chicks were obtained from the Poultry Science, and kept in *Coccidia* and pathogen-free rooms, in disinfected wire-floor cages and provided feed and water *ad libitum*. Each bird was tagged with a leg band and weighed prior, during and at the end of each study. Weight gain/loss was calculated by subtracting the initial weight before inoculation from the weight obtained following challenge and before termination.

#### Cyclophosphamide Immunosuppressed Birds

Immunodeficient birds were utilized in order to investigate fine pathological differences between the normal or aberrant organisms. Neonate (1-day-old) Rhode Island Red chicks were intramuscularly (IM) administered with 4 mg (100 mg/kg) cyclophosphamide (CY, Sigma Chemical Co., St. Louis, MO, USA) for 4 consecutive days, in order to suppress B and T cells and immune responses [3]. T cells may partially recover after one month; however, B cells’ suppression can last for several months.

### 2.3. Preparation of Organisms (Oocysts)

Mature organisms (sporulated oocysts) of *E. tenella* originally were obtained from Eli Lilly and Co. (Indianapolis, IN, USA). Fresh cultures were prepared from the ceca of donor birds one week after oral inoculation. The contents were homogenized and the immature organisms were separated from debris by sieve and centrifuged at 400× *g* and sediments added into 2.5% aqueous potassium dichromate to obtain mature normal organisms. In order to induce aberrant organisms, the homogenate containing immature (unsporulated) organisms were cleaned in a solution of 2.5% sodium hypochlorite, and rinsed with distilled water (2×). The homogenates were centrifuged for 10 min at 400*× g* in a saturated salt solution (9:1:*v.v*), then rinsed in distilled water and cultured in 2.5% aqueous potassium dichromate solution to enhance maturation. Organisms were declared mature when at least 90% had reached maturation (sporulated). The organisms were enumerated using a hemocytometer and diluted into PBS according to the required numbers per each experiment before organisms gavaged directly into crop. Normal control animals received sham (PBS) treatment by gavage.

**Scheme 1:**
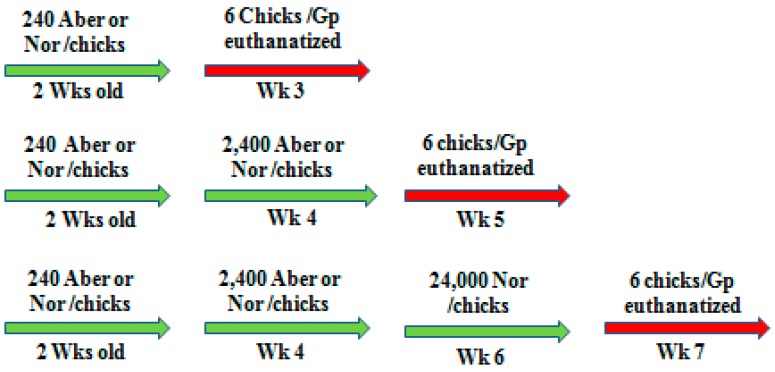
Schematic timeline immunization for 2 strains of chicks (New Hampshire and Rod Island Red) with aberrant (Aber) or normal (Nor) organisms and challenge with highly pathogenic dose (24,000) of normal organisms.

At 2–6 weeks of age, animals were gavage inoculated via crop respectively with any dose of 240, 300, 450, 2400, or 24,000 mature organisms of either normal or aberrant strains (Scheme 1). Each experimental animal received one dose at a time and up to three doses with 2 weeks interval according the experimental design. Infected animals (New Hampshire, Rhode Island Red) were kept in isolation wired cages into separate rooms and provided with food and water *ad lib*. Cages were daily cleaned and disinfected. 

### 2.4. Blood and Tissue Collection

One week after the last challenge dose of inoculum, animals were weighed, and blood was collected into syringes from the wing basilic vein using 25 gauge needles. Animals were humanely euthanatized by cervical dislocation immediately followed by cardiac puncture and tissues collected. Blood was centrifuged and serum aliquots were stored at −20 °C until used. Intestines were immediately removed and examined and a portion of cecal tissues fixed in buffer formalin solution. Slide smears were prepared from the rest of cecal tissues to detect the organisms.

#### Histopathological Scoring

Histopathological slides were prepared from 6 animals/each group and sections stained with Hematoxylin and Eosin. Slides were numerical-labeled (coded) at the pathology lab during the automated staining process. Every field on each pathological slide was thoroughly examined for changes and possible damage. Cecal lesions were scored from 0 to 5 according to the severity of infection [3,18].

0 = Normal mucosa, and negative cecal smears.

1 = No detectable pathology but organisms detected in the smears.

2 = Scattered petechia on mucosa, organisms present on the smear, normal cecal contents.

3 = Focal inflamed and thickened mucosa, some hemorrhage in lumen.

4 = Multifocal inflammation and thickened mucosa, extensive hemorrhage in the lumen, with little or no fecal contents, weight loss.

5 = Severe inflammation and necrosis, enlarged ceca with blood or sloughed off mucosa, moribund or dead birds.

Slides were decoded after completion of pathological evaluation.

### 2.5. Maturation of Organisms (Oocysts Sporulation)

Maturation was determined via microscopic examination. Representative samples were examined and percentage of sporulated organisms determined every 4 h. Maturation was achieved when 90% or more of organisms had completed developmental stages.

### 2.6. Enzyme Linked Immosorbent Assay (ELISA)

The indirect ELISA technique was performed using a soluble oocyst antigen of *E. tenella* according to [3,18,19]. Each serum sample was heat inactivated at 56 °C for 30 min before use and diluted in two-fold increments. Peroxides conjugated Ɣ chain rabbit anti-chicken IgG (Cappel Laboratories, West Chester, PA, USA) was dispensed into each micro-well coated with 11 µg of antigen, and incubated with o-phenylenediamine (Eastman Kodak Co., Rochester, NY, USA). Optical densities were read at 490 nm on ELISA reader (DYnatech Lab Inc., Alexandria, VA, USA).

### 2.7. Experimental Design

In the first experiment, 2-weeks-old and 3-weeks-old inbred CY-immunodeficient-animals were inoculated with 300 and 450 organisms, respectively, of either normal or aberrant form. To compare the immunogenic efficacy, 3-weeks-old inbred New Hampshire or Rhode Island Red animals received 240 and 2400 oocysts of normal or aberrant organisms at a two-week interval. Then, animals were challenged with a high pathogenic dose (24,000) of normal organisms*.* One week after each inoculation, six animals from each group were weighed and bled, then euthanized to collect samples for pathological investigations.

### 2.8. Frozen Sections

Organisms were centrifuged in 20% (*w*/*v*) bovine serum albumin (BSA) and 15% (*w*/*v*) sucrose. The pellets were fixed with 2 drops/mL of 25% glutaraldehyde. Sections were quick frozen in liquid nitrogen and blocks were sectioned at the 18 µm setting on cryostat. Organisms were embedded with mounting agent to protect from shattering during the sectioning procedure. The sections were stained with Hematoxylin and Eosin.

### 2.9. Electron Micrographs

The ultrastructural modifications of aberrant and normal organisms were investigated during maturation. Therefore, organisms were embedded in cross-linked BSA media and frozen in liquid nitrogen prior to cryostat sectioning at 16 µm. These sections were directly immersed in Carnovsky fixative, re-embedded in BSA, post fixed in osmium tetroxide and embedded in osmium tetroxide and finally embedded in Spurr’s Plastic. The blocks were sectioned with KLB ultramicrotome 111 with a diamond knife. The sections were stained with uranyl acetate and lead citrate and examined with Ziess EM Transmission Electron Microscope (TEM) and micrographs were developed.

### 2.10. Statistical Analysis

Parameters were analyzed with Prism 6 software (Graph Pad Software, Inc., La Jolla, CA, USA). Data are expressed as mean ± SEM unless otherwise stated. One hundred organisms were monitored each time from 3 different samples. Maturation of organisms was calculated when a minimum of 90% of organisms formed infective sporozoites. Weight gain/loss was calculated by subtracting the initial weight before inoculation from the weight obtained following each challenge inoculation and before termination. Statistical analysis was performed utilizing a two-way analysis of variance (ANOVA) followed by Bonferroni multiple comparison post hoc test for comparisons. Statistical significance was set at *p* ≤ 0.05.

## 3. Results

### 3.1. Maturation and Ultrastructure

**Figure 1:**
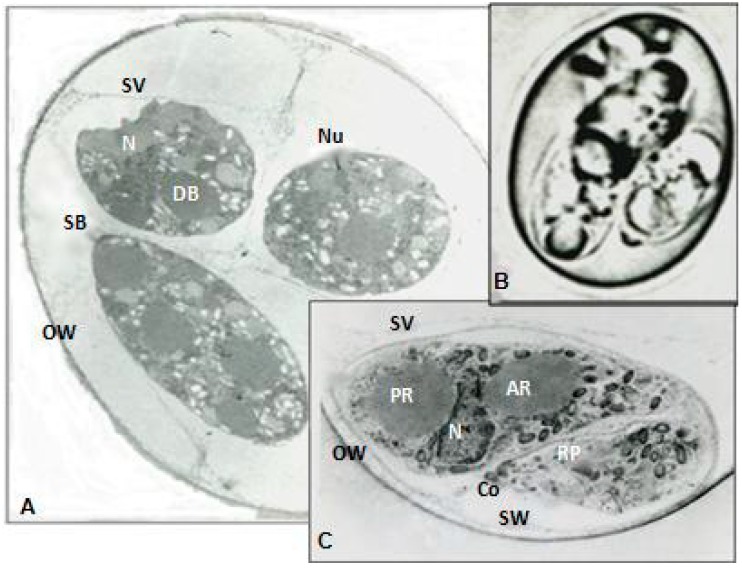
(**A**,**C**) Transmission electron micrographs (TEM) from mature (**A**,**C**) and light micrograph (**B**) from normal organism representative (*E. tenella*). (**A**) Mature normal organism (oocyst) representative 34 h of developmental stage (sporulation) demonstrating 3 of 4 sporocysts, oocyst wall (OW), Stieda body (SB), sporocysts wall (SW), sporocysts veil (SV) dividing the organism into a honey-combed chamber, nucleolus (Nu) ×7000. (**B**) A normal organism under light microscopy with 3 of 4 sporocysts ×800. (**C**) Enlarged micrograph from mature sporocyst contains 2 mature sporozoites within the sporocyst’s wall 34 h of developmental stage. Each sporozoite contains refractile bodies ×13,000. Note Nucleus (N), Dense body (DB), Anterior (AR) and posterior refractile bodies (PR), Rhoptry (Rh), nucleolus (Nu), Sprocyst veil (SW), Oocyst wall (OW).

Ultrastructural modifications during developmental maturation were investigated to compare aberrant and normal organisms utilizing transmission electron micrographs. Normal organism development was completed after 34 h of maturation (sporulation) when they contained 4 fully formed sporocysts each to shield 2 infective sporozoites within. Sporocysts walls and sporocysts veil ultrastructures visible by TEM (Figure 1A,B) were required to sustain their infectivity, as will be presented. In contrast, aberrant organism maturation was more sluggish and about 23% longer than normal organisms (44 h vs*.* 34 h).

**Figure 2:**
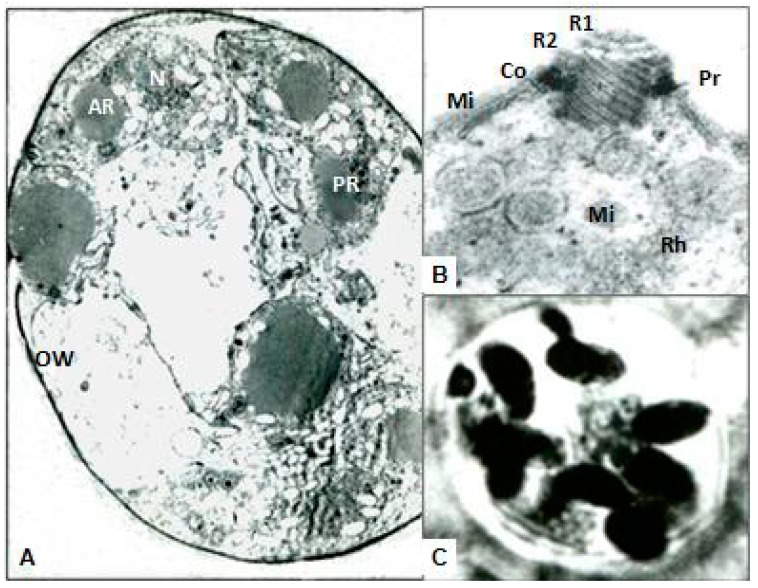
(**A**–**C**) Mature aberrant organism. (**A**) Transmission electron micrograph (TEM) from mature aberrant organism representative contains free sporozoites. Micrograph demonstrates section through a mature aberrant organism 44 h after development. Unlike normal, aberrant organisms lack sporocysts, sporocysts wall and veil and stieda body as evidenced. ×7000. (**B**) High magnification of the anterior end from free sporozoite showing conoid (Co), piercing organelles, preconoidal rings (R), polar ring (Pr), and microneme (Mi). ×11,600. (**C**) Cryostat section stained with Hematoxylin and Eosin of mature aberrant organism demonstrates 8 free sporozoites, polar granule and 2 masses of oocyst residuum and lack of sporocyst structure. Light microscopy ×790.

Aberrant organisms had free naïve sporozoites within the oocysts confinement without any protective sporocysts walls and veils (Figure 2A–C) dissimilar to normal ones. Further, aberrant organisms had fragile outer membranes (oocyst wall) which disrupted in occasions during TEM process for ultrastructure investigation. In addition, aberrant organisms contained 2 masses of rudimentary organelles as residuum of sporocysts (Figure 1C). However, normal and aberrant organisms both had an equal number of (8) banana-shape-sporozoites. Aberrant sporozoites similarly contained nucleus, rhoptries, conoidal piercing organelles, and polar rings and fibrillate ultrastructures required for invasion and lodging into gut epithelial cells. Otherwise, aberrant organisms appeared to have similar microstructures to normal ones in shape (round to oval), measurements (25 to 35 µm by 22.5 to 27.5 µm) and the size of each banana-shaped free sporozoite (12.5–14 µm by 2.5 µm). Over 90% of immature organisms processed to mature into the aberrant form, compared to 95% of normal organisms. This study proved the specific protective effects of the sporocysts provided for the longevity and infectivity of the sporozoites.

### 3.2. Immunodeficiency and Comparative Pathogenicity

**Figure 3:**
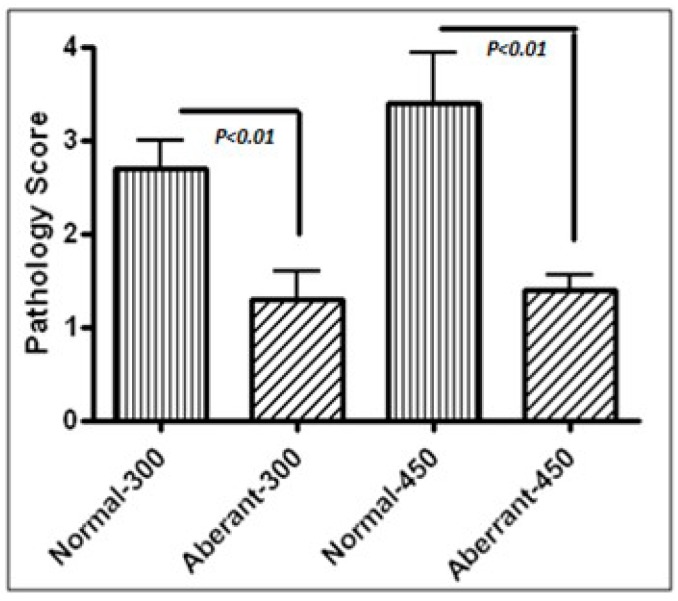
Pathological scores from Cylophosphamide (CY)-immunodeficient animals inoculated with 300 (at 2 weeks of age) or 450 (at 3 weeks of age) of either mature (sporulated) aberrant or normal organisms. Samples are collected 1 week after initiation of infection by oral gavage. Statistically significant pathological damages were detected in animals treated with normal organisms compared to aberrant forms (*p* < 0.01). As expected no antibody production was detected in sera from these CY-immunodeficient animals.

An induced immunodeficient model was utilized to compare the fine detailed pathogenecity of aberrant and normal organisms. Neonate chicks were exposed to cyclophosphamide treatment for 4 consecutive days, compared to the sham-treatment in immune-intact animals. CY-treated-immunodeficient animals had extensive stunt development as appeared with bare skin and patchy feather growth. Respectively, at 2 or 3 weeks-old, CY-immunodeficient-juvenile chicks were gavage inoculated with low doses of mature (2 weeks with 300 or 3 weeks with 450) normal or aberrant organisms. Animals treated with normal organisms became moribund and developed severe GI inflammatory responses and bloody diarrhea, and gained significantly less weight (Figure 3, Table 1). In contrast, CY-immunodeficient-animals tolerated aberrant organisms with significantly more weight gain and pathological lesions (*p* < 0.001). As was expected, CY-immunodeficient animals developed no detectable (protective) antibodies in contrast to immune-intact animals. In gross pathology, CY-immunodeficient animals had rudimentary thymus, splenic and bursa of Fabricius structures (data not shown) due to the cytotoxic effect of CY on B and T cell formation. Yet, immunodeficient animals treated with normal organisms presented significantly severe pathological damages to the gut than those treated with aberrant forms. These data support that aberrant organisms are significantly less invasive and less pathogenic than normal forms (Figure 3, Table 1). All normal animals were protected against challenge doses with no visible intestinal lesions or organisms detected from their intestinal smears. In contrast, all CY-immunodeficient animals were unprotected against low doses of challenge infection (data not shown).

### 3.3. Organisms and Protective Immunogenicity

There is a strain variation in immune response against organisms amongst chicks. Therefore, 2 different inbred strains, Rhode Island Red and New Hampshire immune-intact animals were utilized for this experiment in order to compare protective immune response against infection. Further, day-old chicks lack immunity but consume their egg yolk which is rich in maternal immunity and become protected against pathogens during the first week of their life. However, in the second week as maternal immunity weans off, chicks become sensitive to infections and start to develop their active immunity. Therefore, two-weeks-old chicks were used to initiate the following investigations as these animals are sensitive to infections yet capable of launching an immune response against pathogens. Two-weeks-old animals were immunized with serial inoculations of aberrant or normal forms (240 and 2400) with 2 weeks interval. All animals were challenged with a high pathogenic dose of normal form (24,000). Those animals immunized with aberrant organisms gained weight more and developed lower cecal pathological lesions than those immunized with normal organisms. These animals were protected against the challenge infection (Table 2, Table 3 and Table 4). Naïve unimmunized animals challenged with a pathogenic dose (24,000 normal organisms) lost weight and developed severe bloody diarrhea and significantly more extensive inflammatory pathological lesions than immunized animals (unimmunized 3.2–4.1 vs. immunized 0–1) (Table 4).

In general, Rhode Island Red animals were found to be more susceptible to disease, as they developed more severe lesions and significantly less weight gain compared to New Hampshire animals (Table 1, Table 2 and Table 3). In addition, naïve Rhode Island Red animals developed significantly more severe pathological lesions (4.1 + 0.3 vs. 3.2 + 0.5 *p* < 0.05) and lost weight more (*p* < 0.01) than naïve New Hampshire animals (Table 4).

Animals immunized with aberrant forms were protected against the challenge infection, gained more weight, developed significantly less or no cecal lesions and more antibodies (except Rhode Island Red), compared to those immunized with normal organisms (*p* < 0.05). Rhode Island Red animals immunized with aberrant organisms developed significantly less lesions compared to those immunized with normal organisms, yet released significantly less Ab than those immunized with normal strains. However, the reason behind this discrepancy is not known. Uninfected control animals remained anti-coccidal antibody negative. There was a minor difference between the lesion scores from immunized animals but it did not reach significance. These investigations proved that the sporocysts are required to preserve infectivity in these organisms. Overall, aberrant organisms were less pathogenic in immunodeficient animals yet protective in immunocompetent animals against the challenge infection.

## 4. Discussion

Coccidiosis causes severe gut inflammation, diarrhea, malabsorption, malnutrition and weight loss. The common preventive practice against coccidiosis in poultry and livestock includes application of antibiotic supplementation into daily diets which contaminates eggs, milk, and meat production. Antibiotics enter the food cycle and are consumed by humans with possible allergies, antibiotic resistance, and other yet unknown side effects. Antibiotic additives include tetracyclines, as commonly used in poultry, and are deposited and remain in bones and meat products. They become a potential human health risk regardless of monitoring appropriate antibiotic withdrawal times before reaching the market [20]. Further, sulfonamide use in the form of anticoccidial additives in food animals has encountered the emergence of drug-resistant strain infections [21]. The Center for Disease Control and Prevention (CDC) has speculated that annually over 2,000,000 sicknesses and 23,000 mortalities are due to microbial resistance to antibiotics in the USA. Recent consumer awareness regarding the antibiotic residue polluting food animal products and antibiotic resistant microbials has created trepidation and specific recommendations to diminish overuse of antibiotics as growth promoters in livestock in the USA [22] and worldwide. However, these recommendations may not be effective or applicable without surrogate implementations such as safe and effective as well as feasible vaccine availability against economically important coccidiosis in the food animal industry. The focus of this investigation was on discovery and establishment of proof-of-concept in basic methodology, for development of aberrant organisms to lead as candidate vaccines in coccidiosis.

Cyclophosphamide is a potent immune-modulating agent discovered in 1959 and ever since has been used in autoimmune diseases and as a chemotherapeutic in cancer. CY is metabolized to active form 4-hydroxy-CY, which spontaneously breaks down to reactive intermediates, phosphoramide mustard and acrolein [23]. Phosphoramide mustard possesses an alkylating property which mediates the anti-proliferative and cytotoxic actions of CY and cross links DNA with mitotic B and T cells. In birds, immunoglobulin synthesis and antibody formation are regulated by Bursa of Fabricius, and CY destroys bursal B cells as well as thymus T cell populations/production [2,3]. After four consecutive days of treating neonate chicks with a cytotoxic agent, CY-treated juvenile animals visibly lacked immune processing organs, thymus and bursa and bared rudimentary splenic structures. CY-immunodeficient animals had pervasive growth stunting and weighed significantly less than immune-intact animals (60%). Therefore, these animals were found to be more susceptible to infections when challenged with a lower dose of organisms and developed more severe lesions than immune-intact animals. Additionally, immunodeficient animals had no detectable antibody titers in their sera even after being exposed with invasive organisms. Immunodeficient animals were challenged with organisms at two or three weeks of age when they lack both active B-cells and T-cells; while recovery of T-cells usually occurs after four weeks and B-cells after 7–12 weeks of age [2,3]. Further, coccidiosis is an immunosuppressive disease which weakens the immune system specifically in juvenile animals. Intestinal pathological scores caused by normal organisms were significantly more severe than those inoculated with aberrant organisms in both experiments. Yet, these animals endured the aberrant organisms proving the concept that altered organisms were significantly of low-pathogenicity even in immunodeficient animals. CY may cause GI effects; however, no attempts were taken to eliminate CY possible side effects on GI tracts except practicing extreme hygiene in animals. It is plausible that some of the pathological findings in these immunodeficient animals could have been provoked by CY more than direct effects from coccidial infection. Nevertheless, CY was similarly used in all immunodeficient animals. Therefore, differences obtained between various groups of immunodeficient animals can be mainly attributed to the pathogenic strength of normal versus aberrant organisms than direct effects from CY on GI tracts.

Strains of chicks with phenotypic differences may express various immunological/pathologic responses and resistance/susceptibility to diseases. In this investigation, two inbred strains, Rhode Island Red and New Hampshire, were chosen in order to compare immune responses against coccidial infection. Differences were detected in weight gain/loss. Naïve Rhode Island Red animals developed more severe lesions compared with New Hampshire animals. New Hampshire animals were significantly more robust and gained weight more and better tolerated challenge infections compared to the Rhode Island Red animals. As expected, uninfected control animals remained anti-coccidal antibody negative, while protective antibody titers were detected in immunized animals with aberrant forms. Yet, both strains became effectively and better protected with aberrant forms against ultimate infectious challenge than those immunized with normal forms. Aberrant organisms provoke immunity without pathogenicity. The pathogenicity after challenge was significantly lower in both groups of animals immunized with aberrant organisms. These included lack of bloody diarrhea and severe weight loss. In addition, the level of Ab production was significantly higher in the New Hampshire group immunized with aberrant, but not in Rhode Island Red animals. It is not clear whether the discrepancy in Ab production is due to immune response differences in animal strains or if other factors may have influenced the results. It is conceivable that higher titers of Ab production may not be protective in some strains of animals as seen in severe cases of infections and autoimmune diseases with unproductive surges of Ab production. These investigations proved that the sporocysts are required to preserve infectivity of the organisms. Overall, both animal strains tolerated better immunization with aberrant forms, gained more weight during immunization and when challenged with high dose pathogenic form compared to those immunized with normal forms, as presented in the results section.

This technique may be applicable to be used in other Apicomplexan organisms. For instance, *Toxoplasma* is another related member of Apicomplexan organisms with severe acute and chronic complications as well as feto-maternal syndrome in humans and animals. *Toxoplasma* has an intra intestinal (coccidian forms) exclusive in cats as a definitive host and in extra intestinal stages is cosmopolitan as it infects intermediate hosts (all animals and man), as well as every single organ and cell in the body [7,24,25,26]. It is conceivable that the same modifications may be applicable for oocysts of *Toxoplasma* to form aberrant organisms for a safe vaccine to immunize cats against toxoplasmosis as a preventive measure to protect humans and animals against disease.

A century after the original discovery of poultry coccidiosis (*Eimeria*), a unique method of altering infectivity of organisms has been achieved, as presented in this investigation. Previous studies had either relied on genetic variation and selection in chicks [27], or altered organisms by exposing them to irradiation [28]. The viable vaccination with Coccivac, which is a mixture of different pathogenic *Eimerias* in chicks, has been used for over 60 years (Edgar 1952) in the USA [15] with several side effects. The pathogenic live vaccine promotes weight loss and poor feed conversion and extends the required time to develop solid protective immunity response to encounter the disease, the possibility of leading to new infections and difficulties in preparation, administration and husbandry of the flocks. Other experimental attempts for vaccine production include attenuated strains and serial passages of *E. tenella* into chorio-allantoic chick embryo [16] which were proved to be ineffective. Our current report is supported by previous findings related to altered organisms from two different pathogenic strains, *E. tenella* and *E. necatrix,* using two different strains of inbred White Leghorn chicks [18]. In this study, the inbred New Hampshire response was in accord with White Leghorns immunized with aberrant organisms with complete protection from challenge infection and diarrhea. This study further exposed the ultrastructures of organisms and proved specific roles for sporocysts walls and veils to sustain pathogenicity and longevity of the infective sporozoites.

## 5. Conclusions 

Overall, aberrant organisms were significantly less pathogenic in immunodeficient animals compared to the normal organisms, yet protective in immunocompetent animals against severe challenges. As proof of concept, lack of protective ultrastructures (sporocysts walls and veils), reduced pathogenicity, including diarrhea and weight loss, and preserved immunogenicity seems appropriate to indicate a potential use for the aberrant organisms to warrant further trials for possible attenuated vaccine production in future investigations.

## Figures and Tables

**Table 1:** Effects of low doses of aberrant and normal organisms in juvenile immunodeficient or immune-intact Rhode Island Red animals.

Gp	Week Old	CY Treated	Organism/	Weight Gain	Ab Titer
1	2	+	300 N	60 ± 3.4	0
2	2	+	300 A	57 ± 5.7	0
3	3	+	450 N	87 ± 7.2 ^b^	0
4	3	+	450 A	106 ± 7	0
5	3	−N *	450 N	95 ± 1.4	50 ± 1
6	3	−N *	0	190 ± 8	0

Cyclophosphamide (CY) immunodeficient (+) or immune-intact (−) chicks were inoculated with a low dose of 300 (2 weeks old) or 450 (3 weeks old) normal or aberrant organisms. Two weeks and three weeks old (CY) immunodeficient animals were chosen when lacking both T and B cells. Data is shown as mean ± SEM. Gp = Group, N = Normal organisms, A = Aberrant organism, N * = Normal Immune-intact birds. Mean Body Weight gain/g. N = 6/group. ^b^ *p* < 0.05.

**Table 2:** Immune-intact animals were immunized with first dose of normal or aberrant organisms at week 1.

Gp	Birds Strain	Strain	Organism/	Weight Gain	Lesion	Ab Titer
1	New Hampshire	A	240	104 ± 5	1 ± 0.1 ^b^	0
2	“	N	240	85 ± 7	1.3 ± 01	0
3	“	C	0	123 ± 7 *	0	0
4	Rhode Island Red	A	240	114 ± 3	1 ± 0.1	0
5	“	N	240	95 ± 2	1 ± 0.1	0
6	“	C	0	190 ± 3 *	0	0

Six birds/group (Gp) were inoculated with Normal (N) or Aberrant (A) organisms (week 1). Data is shown as mean ± SEM. Normal control (C) birds received (0) sham inoculation. Lesion scores. Mean Body Weight gain/g. N = 6/group. ^b^ *p* < 0.05. * *p* < 0.01.

**Table 3:** Animals immunized with second dose of normal or aberrant organisms. Six/group animals which were immunized with 240 and 2400 Normal (N) or Aberrant (A) organisms (week 4) with 2-weeks interval. Control (C) birds received (0) sham inoculation.

Gp	Bird Strain	Strain	Organism/	Weight Gain	Lesion	Ab Titer
1	New Hampshire	A	2400	133.4 ± 6	1 ± 0.1	58.0 ± 2 ^b^
2	“	N	2400	133.4 ± 3	1 ± 0.1	50.0 ± 1
3	“	C	0	247.4 ± 2	0	0
4	Rhode Island Red	A	2400	152.3 ± 1 *	1 ± 0.2 ^b^	25.0 ± 2 *
5	“	N	2400	104.9 ± 2	1.4 ± 0.1	17.0 ± 1
6	“	C	0	218.9 ± 5	0	0

Data is shown as mean ± SEM. Lesion scores. Mean Body Weight gain/g. N = 6/group. ^b^ *p* < 0.05;* p < 0.01.

**Table 4:** Immunized animals with two different doses of normal or aberrant were challenged at 6 weeks of age with high numbers of infective normal organisms.

GP	Bird Strain	Strain	Organism/	Weight Gain	Lesion	Ab Titer
1	New Hampshire	A	24,000	100 ± 2 *	0 ^b^	69 ± 4 ^b^
2	“	N	24,000	50 ± 3	0.35 ± 0.1	63 ± 2
3	“	N *	24,000	−45 ± 2 ^#^	3.2 ± 0.5	50 ± 3
4	“	C	0	247 ± 5	0	0
5	Rhode Island Red	A	24,000	72 ± 3	0.76 ± 0.1 ^b^	44 ± 6 ^b^
6	“	N	24,000	64 ± 3	1 ± 0.2	81 ± 2
7	“	N *	24,000	−80 + 4 ^#^	4.1 ± 0.3	ND
8	“	C	0	219 ± 7	0	0

Six birds/group were immunized with 240 and 2400 Normal (N) or Aberrant (A) organisms (weeks 2 and 4) with 2-weeks interval. Control (C) animals received (0) sham inoculation. Infected control group (N *) received no previous immunization before final challenged inoculum. Lesion scores. Mean Body Weight gain/g significant. ND = Not done. ^b^ *p* < 0.05; * p < 0.01; ^#^ *p* < 0.001.

## Abbreviations

**CY**, Cyclophosphamide; **TEM**, transmission electron microscope; ***E.***, *Eimeria t*; **H&E**, Hematoxylin and Eosin; **ELISA**, Indirect Enzyme Linked Immunosorbant Assay.
